# Responsiveness to Mental Health Needs across European countries

**DOI:** 10.1192/j.eurpsy.2025.98

**Published:** 2025-08-26

**Authors:** C. Arango

**Affiliations:** Hospital General Universitario Gregorio Marañon, UCM, CIBERSAM, Madrid, Spain

## Abstract

**Abstract:**

This study evaluated European countries’ responsiveness to mental health (MH) needs by analyzing key performance indicators (KPIs) across healthcare, workplaces, schools, and society. Data from the 2023 Headway Initiative included 15 KPIs related to healthcare responsiveness—such as workforce availability, facilities, quality of care, and MH expenditure—and 14 KPIs assessing responsiveness in workplaces, schools, and society. KPI scores were standardized on a 1-10 Likert scale, with higher scores indicating better performance. Bivariate correlations examined associations between responsiveness and overall MH status. Sweden (10.0), Denmark (8.8), and Finland (8.3) demonstrated the highest responsiveness in healthcare, whereas Romania (1.0), Slovakia (1.1), and Latvia (1.2) ranked lowest. In non-healthcare domains, Germany (10.0), France (9.1), and Denmark (9.1) were the most responsive, while Greece and Slovakia (1.0) exhibited the poorest performance. These findings highlight significant disparities in MH support across Europe and emphasize the need for policies that address inequalities and bridge treatment gaps. Future efforts should focus on balancing MH status improvements with sustained investment in MH responsiveness.

**Disclosure of Interest:**

C. Arango Speakers bureau of: Dr. Arango has been a consultant to or has received honoraria or grants from Abbot, Acadia, Ambrosetti, Angelini, Biogen, BMS, Boehringer, Carnot, Gedeon Richter, Janssen Cilag, Lundbeck, Medscape, Menarini, Minerva, Otsuka, Pfizer, Roche, Sage, Servier, Shire, Schering Plough, Sumitomo Dainippon Pharma, Sunovion, Takeda and Teva.

